# Protocol for visualizing microtubules in transzonal projections of mouse ovarian follicles

**DOI:** 10.1016/j.xpro.2026.104924

**Published:** 2026-10-28

**Authors:** Takao Tsurumaki, Akihiro Aikawa, Masamitsu Sato, Mika Toya

**Affiliations:** 1 Laboratory of Cytoskeletal Logistics, Department of Life Science and Medical Bioscience, Graduate School of Advanced Science and Engineering, Waseda University, Shinjuku-ku, Tokyo, Japan; 2 Institute for Medical-Oriented Structural Biology, Waseda University, Shinjuku-ku, Tokyo, Japan; 3 Laboratory for Histogenetic Dynamics, Graduate School of Pharmaceutical Sciences, Kyoto University, Sakyo-ku, Kyoto, Japan

**Keywords:** Cell Biology, Cell-based Assays, Microscopy

## Abstract

Visualizing microtubules within tissues remains technically challenging, limiting our understanding of their organization and function *in vivo*. Here, we present a protocol for visualizing microtubules in transzonal projections (TZPs), cellular projections that connect ovarian somatic cells and oocytes within mouse ovarian follicles. We describe steps and conditions for ovarian fixation, cryosection preparation, and immunostaining to preserve fine microtubule structures. We then detail procedures for super-resolution imaging to visualize fine microtubules within TZPs.

For complete details on the use and execution of this protocol, please refer to Aikawa et al.^1^

## Highlights

• Optimized fixation preserves microtubules in mouse tissues
• Visualizes microtubules in transzonal projections of mouse ovarian follicles
• Combines cryosectioning, immunostaining, and super-resolution imaging
• Applicable to microtubule visualization in other mouse tissues

## Abstract (editor highlights)

Publisher’s note: Undertaking any experimental protocol requires adherence to local institutional guidelines for laboratory safety and ethics.

## Graphical abstract

**Figure:**
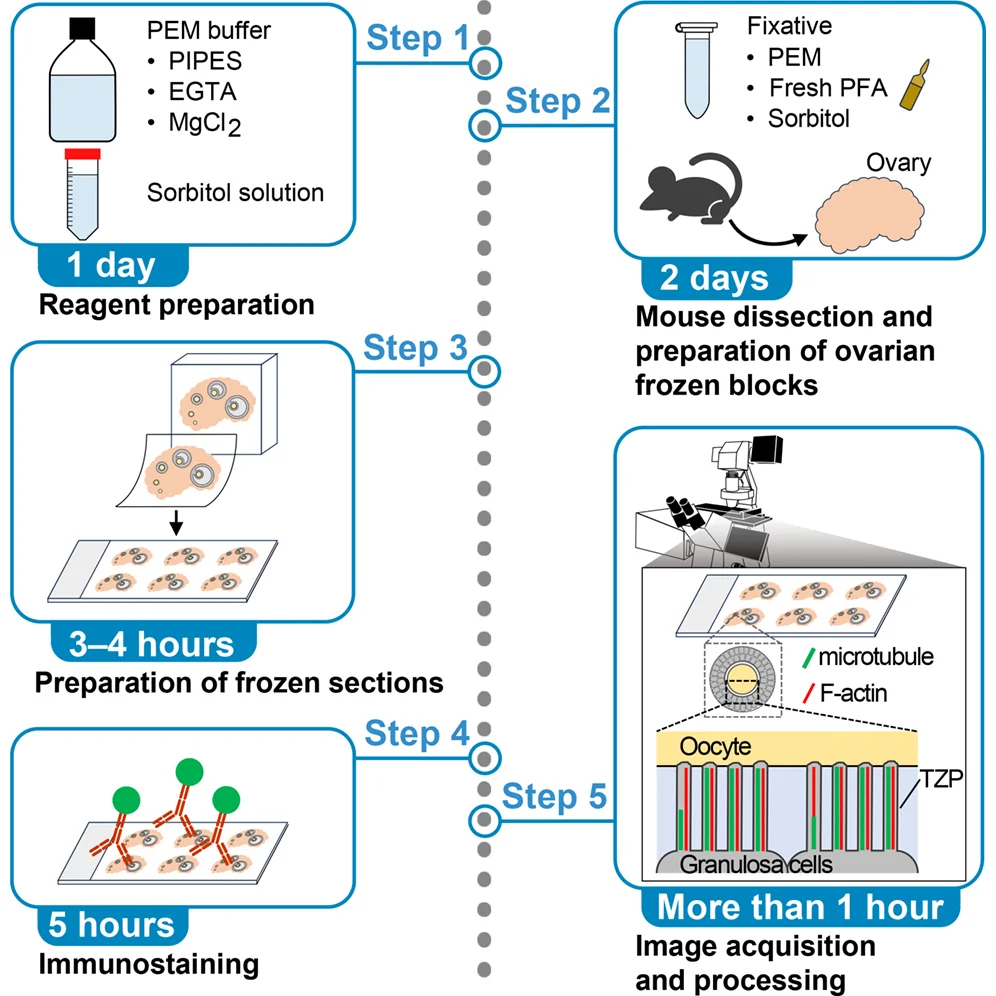

## Before you begin

Although this protocol describes detailed procedures for fixation, immunofluorescent staining, and imaging of microtubules in transzonal projections (TZPs) within mouse ovarian follicles, it can also be applied to visualize microtubules in other tissues, such as intestinal and kidney epithelium.^2^^,^^3^ For these tissues, cut the intestine into approximately 1 cm segments and bisect the kidney longitudinally before fixation to ensure adequate penetration of the fixative. Appropriate fixation is critical for preserving microtubules, whereas high-resolution imaging and z-stack acquisition are important for detecting the fine microtubules within TZPs, which generate weaker signals than prominent cytoplasmic microtubule arrays.

### Innovation

Microtubule visualization in intact tissues is challenging because microtubules are highly dynamic and sensitive to fixation conditions. This protocol integrates optimized fixation, buffering, and tissue preparation procedures into a reproducible workflow that preserves microtubule architecture in vivo. The method enables sufficient visualization of microtubules within transzonal projections (TZPs) of mouse ovarian follicles, revealing microtubules in the majority of TZPs, in contrast to previous reports that detected them only infrequently.^4^^,^^5^^,^^6^ In addition, the underlying fixation and staining strategy has been successfully applied to multiple epithelial tissues, including the intestine, kidney, and ovary, demonstrating its broader utility for visualizing tissue microtubules.^1^^,^^2^^,^^3^

### Institutional permissions

All animal procedures described in this protocol were conducted in accordance with institutional ethical guidelines and approved by the Animal Review Committee of Waseda University (approval numbers A24-082, A24-083, A25-078, A25-079, A26-060, and A26-061). Mice were maintained and bred under specific pathogen-free (SPF) conditions. All animal experiments must be approved by the appropriate institutional and/or national animal ethics committees and performed in accordance with relevant guidelines and regulations.

### Reagent preparation

**Timing: 1 day**

1. Prepare 4× PEM buffer (200 mL).
  a. Add 24.18 g PIPES, 1.52 g EGTA, 0.81 g MgCl_2_·6H_2_O to 150 mL Milli-Q water in a beaker.
    ***Note:*** PIPES will dissolve after KOH is added in the next step.
  b. Add approximately 8 g KOH.
    **CRITICAL:** KOH is a supplied as pellets, so it may not be possible to weigh exactly 8 g. Do not exceed 8 g at this stage, as excess KOH may raise the pH above 6.9.
  c. Allow the solution to cool to 25°C.
  d. Adjust the pH to 6.9 using 2 M KOH.
    ***Note:*** Approximately 3 mL of 2 M KOH is required.
  e. Bring the final volume to 200 mL with Milli-Q water.
  f. Store the buffer in an autoclaved bottle at 4°C for up to one month.
    **CRITICAL:** Use PEM buffer throughout fixation and staining. Substitution with phosphate-buffered saline may result in reduced preservation of microtubules.
2. Prepare additional buffers and fixative as described in materials and equipment.
  a. Saline solution.
  b. 1× PEM.
  c. 10% Triton X-100.
  d. 1M sorbitol.
  e. 2% formaldehyde/50 mM sorbitol in PEM (fixative).
  f. 15%, 20%, and 30% (w/v) sucrose solutions.
  g. 0.1% Triton X-100 in PEM.
  h. 0.2% Triton X-100 in PEM.
  i. 3% BSA/0.1% Triton X-100 in PEM.

**CRITICAL:** Prepare 2% formaldehyde/50 mM sorbitol in PEM (fixative) immediately before ovary dissection.

**CRITICAL:** Including 50 mM sorbitol in the fixative improves preservation of microtubules.

**CRITICAL:** For optimal preservation of microtubules, prepare the fixative from a freshly opened aliquot of formaldehyde solution (typically supplied as 16% formaldehyde solution in sealed glass ampoules). In our experience, previously opened aliquots can still produce acceptable staining for preliminary experiments, but freshly opened aliquots provide the most consistent results for quantitative analyses and image acquisition.

## Key resources table

**Table:** 

REAGENT or RESOURCE	SOURCE	IDENTIFIER
**Antibodies**		

Mouse anti-α-tubulin-FITC (dilution 1:200)	Sigma	F2168; RRID: AB_476967

**Chemicals, peptides, and recombinant proteins**		

Alexa FluorTM 647 Phalloidin (dilution 1:400)	Thermo Fisher	A30107
DAPI solution (dilution 1:1000)	Dojindo	340-07971
Sodium Chloride	FUJIFILM Wako	192-13925
D-Glucitol (D-Sorbitol)	Nacalai tesque	32021-95
PIPES	Dojindo	345-02225
Magnesium Chloride Hexahydrate	FUJIFILM Wako	135-15055
EGTA	Nacalai tesque	15214-92
TritonX-100	Nacalai tesque	12967-32
Potassium Hydroxide	FUJIFILM Wako	166-21811
16% Formaldehyde Solution	Thermo Fisher	28908
Sucrose	FUJIFILM Wako	196-00015
Albumin, from Bovin Serum, Globulin Free	FUJIFILM Wako	015-15103
Tissue-Tek O.C.T. compound	Sakura Finetek	45833
Prolong Glass	Thermo Fisher	P36980
Immersol 518 F/30°C	Zeiss	N/A

**Experimental models: Organisms/strains**		

C57BL/6NCrSlc wild-type mice, sex: female, age: 3–5 weeks	Sankyo Labo Service	N/A

**Software and algorithms**		

ZEN	Zeiss	https://www.zeiss.com/microscopy/en/products/software/zeiss-zen.html RRID:SCR_013672
Imaris	Oxford instrument	https://imaris.oxinst.com/ RRID: SCR_007370

**Other**		

Nunc Multidish 24	Thermo Fisher	142475
Peel-A-Way Disposable Embedding Mold (Truncated-T12)	Polysciences	18986-1
MAS coat slide glass white	Matsunami Glass	MAS-01
Precision cover glass #1.5H 24×50 mm	THORLABS	CG15KH1
High-Profile Disposable Blade DB80HS	Leica	14035843490
A-PAP PEN	Daido Sangyo	N/A
Slide mailer	Bio Medical Science	BC-68951
Incubation Chamber for 10 slides Dark Orange	Cosmo Bio	10DO
SteREO Descovery.V12	Zeiss	N/A
Cryostat	Leica	CM1950
LSM980 laser scanning confocal microscope with Airyscan 2	Zeiss	N/A

***Note:*** To achieve optimal image quality, use a mounting medium and coverslip with refractive indices matched to the immersion oil. We recommend using the regents listed in the key resources table.

## Materials and equipment

**Table:** 4× PEM

Reagent	Final concentration	Amount
PIPES	0.4 M	24.18 g
EGTA	20 mM	1.52 g
MgCl_2_·6H_2_O	20 mM	0.81 g
KOH	N/A	< 8 g
2M KOH	To pH 6.9	As required
Milli-Q	N/A	Complete volume to 200 mL
**Total**	**N/A**	**200 mL**

Store at 4°C for up to one month.

**Table:** Saline solution

Reagent	Final concentration	Amount
Sodium Chloride	0.9%	0.9 g
Milli-Q	N/A	Complete volume to 100 mL
**Total**	**N/A**	**100 mL**

Store at 20–25°C.

**Table:** 1× PEM

Reagent	Final concentration	Amount
4× PEM	N/A	50 mL
Milli-Q	N/A	150 mL
**Total**	**N/A**	**200 mL**

Store at 4°C for up to one month.

**Table:** 10% Triton X-100

Reagent	Final concentration	Amount
Triton X-100	10%	5 mL
Milli-Q	N/A	Complete volume to 50 mL
**Total**	**N/A**	**50 mL**

Store at 20–25°C.

**Table:** 1 M sorbitol

Reagent	Final concentration	Amount
D-sorbitol	1 M	9.11 g
Milli-Q	N/A	Complete volume to 50 mL
**Total**	**N/A**	**50 mL**

Store at 4°C.

***Note:*** Because sorbitol is fine powder that dissolves relatively slowly at high concentrations, it is convenient to prepare the 1 M stock solution in a 50-mL conical tube. Add the sorbitol powder to prewarmed Milli-Q water and mix thoroughly by shaking until completely dissolved.

**Table:** 2% formaldehyde/50 mM sorbitol in PEM (fixative)

Reagent	Final concentration	Amount
4× PEM	N/A	500 μL
16% Formaldehyde solution	2%	250 μL
1 M sorbitol	50 mM	100 μL
Milli-Q	N/A	1150 μL
**Total**	**N/A**	**2 mL**

Prepare fresh on the day of use and keep at 4°C until use.

**CRITICAL:** Formaldehyde is toxic and carcinogenic. Wear appropriate gloves and handle formaldehyde in a chemical fume hood. Dispose of aqueous solutions containing formaldehyde in accordance with institutional hazardous chemical waste disposal guideline.

**CRITICAL:** For optimal microtubule preservation, prepare the fixative immediately before use from a freshly opened aliquot of formaldehyde solution.

***Note:*** The fixative described above is for a single sample. The following reagents can be stored at 4°C and used for multiple experiments.

**Table:** 15%, 20%, and 30% (w/v) sucrose solutions

Reagent	Final concentration	Amount
Sucrose	15%, 20%, or 30%	7.5 g, 10 g, or 15 g
1× PEM	N/A	Complete volume to 50 mL
**Total**	**N/A**	**50 mL**

Store at 4°C.

**Table:** 0.1% Triton X-100 in PEM

Reagent	Final concentration	Amount
4× PEM	N/A	75 mL
10% Triton X-100	0.1%	3 mL
Milli-Q	N/A	Complete volume to 300 mL
**Total**	**N/A**	**300 mL**

Store at 4°C.

**Table:** 0.2% Triton X-100 in PEM

Reagent	Final concentration	Amount
4× PEM	N/A	3.75 mL
10% Triton X-100	0.2%	0.3 mL
Milli-Q	N/A	Complete volume to 15 mL
**Total**	**N/A**	**15 mL**

Store at 4°C.

**Table:** 3% BSA/0.1% Triton X-100 in PEM

Reagent	Final concentration	Amount
BSA	3%	0.45 g
4× PEM	N/A	3.75 mL
10% Triton X-100	0.1%	0.15 mL
Milli-Q	N/A	Complete volume to 15 mL
**Total**	**N/A**	**15 mL**

Store at 4°C.

## Step-by-step method details

### Mouse dissection and preparation of ovarian frozen blocks

**Timing: 2 days**

This section describes the procedure for excising and fixing mouse ovaries and preparing frozen tissue blocks. The use of PEM buffer throughout fixation and processing helps preserve microtubule architecture and enables visualization of microtubules within the cellular projections such as TZPs.

1. Euthanize the mouse.

***Note:*** This protocol was developed using young (3–5 weeks old) female mice.

2. Excise ovaries from the abdominal cavity of the mouse.
  a. Place the mouse on its back and moisten the abdomen with saline.
  b. Make an incision across the abdomen just below the xiphoid process using scissors.
  c. Excise the ovaries by dissecting them away from the surrounding tissues and cutting the uterus near the fallopian tubes.
3. Quickly remove the uterus and surrounding fat on a paper towel under a stereomicroscope.

***Note:*** Although dissection in saline is preferable to prevent tissue drying, handling is easier on a paper towel.

**CRITICAL:** Remove as much fat tissue as possible. Excess fat may prevent the ovaries from sinking during sucrose infiltration.

**CRITICAL:** Perform ovary dissection rapidly and transfer the samples to fixative as soon as possible to minimize microtubule depolymerization.

4. Transfer the ovaries to a 2-mL tube containing 2 mL of freshly prepared 2% formaldehyde/50 mM sorbitol in PEM.
5. Gently rotate the tube on a shaker for 1 h at 20–25°C.

**CRITICAL:** Perform fixation at 20–25°C. Fixation at 4°C may compromise microtubule preservation.

6. Remove the fixation buffer and add 2 mL of 1× PEM. Gently shake on a shaker for 10 min at 20–25°C. Repeat this wash step two more times.
7. Replace the wash buffer with 2 mL of 15% sucrose solution and gently rotate at 4°C for 2–3 h until the samples sink.
8. Replace the 15% sucrose solution with 2 mL of 20% sucrose solution and gently rotate for approximately 12 h at 4°C.
9. Replace the 20% sucrose solution with 2 mL of 30% sucrose solution and gently rotate at 4°C until the samples sink.

***Note:*** Samples usually sink within 5 h during this step.

10. Equilibrate the ovaries in O.C.T. Compound before embedding.
  a. Fill four wells of a 24-well plate approximately halfway with O.C.T. Compound.
  b. Remove the 30% sucrose solution from the 2-mL tube and transfer the ovaries into the O.C.T. Compound.
  c. Gently stir the samples in the O.C.T. Compound with a spatula for 15 min in each well, transferring them sequentially to the next well every 15 min (60 min total).

**CRITICAL:** Stir gently and avoid generating bubbles or foam.

11. Embed the samples.
  a. Fill embedding molds to approximately 70–80% of their volume with O.C.T. Compound.
  b. Transfer the ovaries into the molds and gently mix with the O.C.T. Compound.
  c. Position each ovary at the bottom center of the mold, with the oviduct oriented horizontally (Figure 1).

**Figure 1:**
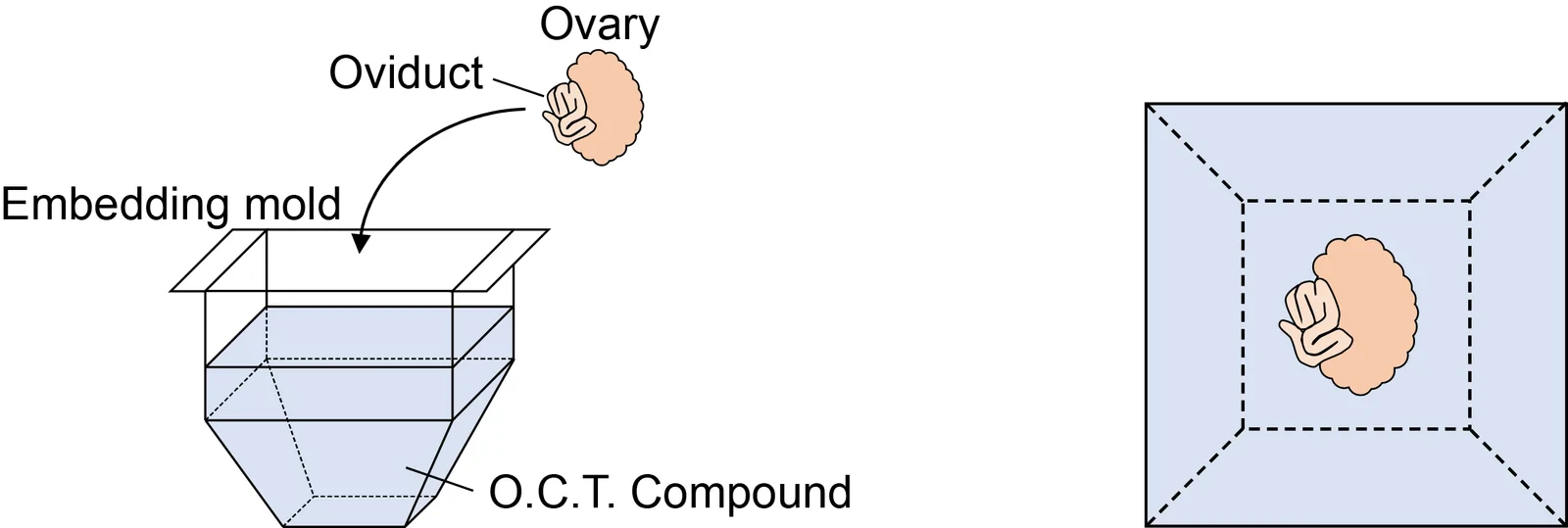
Schematic illustration of ovary placement in the embedding mold Position the ovary in the embedding mold with the oviduct on the lateral side.

**CRITICAL:** Embed each ovary in a separate mold.

12. Freeze the samples.
  a. Pour approximately 5 cm depth of liquid nitrogen into a polystyrene foam container.
  b. Place the embedding mold in a metal mesh basket and immerse it in liquid nitrogen until the O.C.T. Compound is completely frozen and turns white.

**CRITICAL:** Wear appropriate cryogenic protective gloves and eye protection when handling liquid nitrogen.

13. Wrap the frozen blocks in aluminum foil and store immediately at −80°C.

### Preparation of frozen sections of mouse ovaries

**Timing: 3–4 h**

This section describes the procedure for preparing frozen ovarian sections.

14. Set the temperature of the cryostat chamber and the sample holder to −20°C.
15. Equilibrate the frozen ovarian blocks to −20°C.

***Note:*** We usually leave the frozen blocks in the cryostat chamber for 2 h.

**CRITICAL:** Do not return blocks that have been equilibrated at −20°C to −80°C for storage, as this may compromise section quality.

16. Cut the frozen blocks into 5-μm-thick sections and mount them onto glass slides.
17. Draw a hydrophobic barrier around the sections using a PAP Pen (Figure 2A).

**Figure 2:**
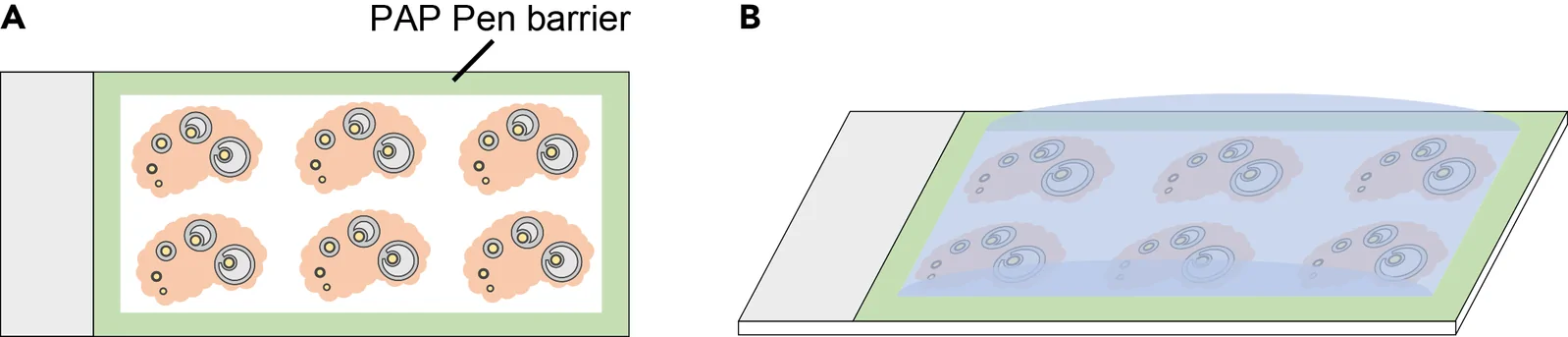
Schematic illustration of PAP pen barrier formation and reagent application (A) Draw a hydrophobic barrier around the ovarian section using a PAP pen. (B) Add the reagent, ensuring that it remains within the PAP pen barrier.
18. Dry the slides for 1 h at 20–25°C with gentle airflow from a fan.
19. Store the slides in a slide box at −80°C until use.

### Immunostaining

**Timing: 5 h**

This section describes the procedure for staining F-actin, microtubules, and DNA in ovarian section.

20. Fill a slide mailer with 0.1% Triton X-100 in PEM. Transfer the slide directly from the freezer into the mailer and gently shake for 10 min.
21. Remove the slide, carefully blot excess moisture with a paper towel, and place it in a humidity chamber.

**CRITICAL:** Remove any residual moisture from the PAP Pen barrier.

22. Gently add 300 μL of 0.2% Triton X-100 in PEM to the section and incubate for 10 min (Figure 2B).

**CRITICAL:** Do not extend the incubation time, as prolonged exposure to 0.2% Tryton X-100 may disrupt tissue morphology and reduce microtubule staining quality.

23. Transfer the slide to a slide mailer containing 0.1% Triton X-100 in PEM and gently shake for 10 min.
24. Remove the slide, carefully blot excess moisture with a paper towel, and place it in a humidity chamber.
25. Gently add 300 μL of 3% BSA/0.1% Triton X-100 in PEM to the section and incubate for 30–60 min.

***Note:*** BSA is used as general blocking regent and is included during antibody incubation to reduce non-specific binding and background fluorescence. This condition is also applicable when the protocol is adapted for conventional two-step immunostaining using other antibodies.

26. Prepare the staining solution during the blocking step.
  a. Prepare 250 μL staining solution per slide by adding Phalloidin Alexa 647 (1:400), FITC-DM1A (1:200), and DAPI (1:1000) to 3% BSA/0.1% Triton X-100 in PEM.

**CRITICAL:** Perform all subsequent steps under light-protected conditions.

***Note:*** This protocol is also compatible with conventional two-step immunostaining using unconjugated primary antibodies and fluorescent secondary antibodies. However, for visualization of the fine microtubule within TZPs, we recommend directly FITC-conjugated DM1A because, in our experience, it provides a higher signal-to-noise ratio and more reliable detection than unconjugated DM1A followed by a fluorescent secondary antibody.

27. Remove the blocking solution, blot excess moisture from the slide, and place it in a humidity chamber.
28. Gently add 250 μL of staining solution to the section and incubate for 2 h at 20–25°C.
29. Wash the slide three times in a slide mailer containing 0.1% Triton X-100 in PEM, with incubation times of 5 min, 10 min, and 15 min, respectively.
30. Remove the slice, blot excess moisture with a paper towel, and place it on a clean laboratory bench.
31. Add one drop of Prolong Glass Antifade Mountant.
32. Carefully place a coverslip onto the slide.
33. Protect the slide from light and leave it at 20–25°C for approximately 12 h, or overnight, until the mountant has completely cured.
34. Observe the slide under a microscope as described in the next section. Although mounted slides can be stored at −20°C for up to 1 week; image quality is generally best when samples are imaged immediately after the mountant has cured.

### Airyscan image acquisition and processing

**Timing: More than 1 h**

This section describes the procedure for acquiring images of TZP microtubules using a confocal microscope.

***Note:*** We utilize an Axio Observer.Z1/7 inverted microscope equipped with an LSM980 confocal scanner and ZEN (Blue edition) software, along with either a Plan-Apochromat 40×/1.3 Oil DIC (UV) VIS-IR M27 objective or a Plan-Apochromat 63×/1.40 Oil DIC M27 objective. Images are detected using a GaAsP-PMT detector. Refer to Tables S1 and S2 for the detailed aquisition settings used to generate the images shown in expected outcomes.

35. Observe the section through the eyepieces and focus on it in Locate mode.
36. Switch the objective lens to the ×40 lens, add a drop of immersion oil and target a follicle.

**CRITICAL:** Select a follicle in which the oocyte is not noticeably distorted and the granulosa cells remain closely associated with the oocyte.

***Note:*** Because TZP microtubules are fine structures and the fluorescence intensity is much lower than that of microtubules in granulosa cells, they may not be readily visible in Locate mode.

37. Set the detection mode to Airyscan SR.
38. Acquire a low-magnification overview image.

***Note:*** The ×40 objective lens cannot clearly capture all TZP microtubules. This imaging is performed to assess the condition of the TZPs in the follicle and to determine which areas should be imaged at higher magnification.

39. Switch to the ×63 lens and add a drop of immersion oil.
40. Using the ×40 image as a guide, select a region of interest containing well-preserved TZPs. (e.g., an intact zona pellucida with granulosa cells remaining closely associated with the oocyte).
41. Enable the “Z-stack” function, then set the step size and thickness.

**CRITICAL:** Typically, a z-stack spanning approximately 2–5 μm with a 0.15 μm interval provides sufficient resolution for visualizing TZP microtubules.

***Note:*** For quantitative analyses, image sections passing near the equatorial plane of the oocyte.

42. Optimize laser power and detector gain while avoiding detector saturation, and then acquire the images.

***Note:*** The detailed image acquisition conditions for the images shown in the expected outcome are shown in Tables S1 and S2.

43. In the Processing tab, select Airyscan Processing, select either 2D SR or 3D Airyscan processing, as appropriate.

## Expected outcomes

Successful application of this protocol should result in clear preservation and visualization of microtubules within mouse ovarian follicles. Ovarian sections stained and imaged according to this protocol reveal microtubules within numerous TZPs, as shown in Figure 3. Compared with conventional approaches, this protocol enables improved preservation and visualization of microtubules within these fine cellular projections.

**Figure 3:**
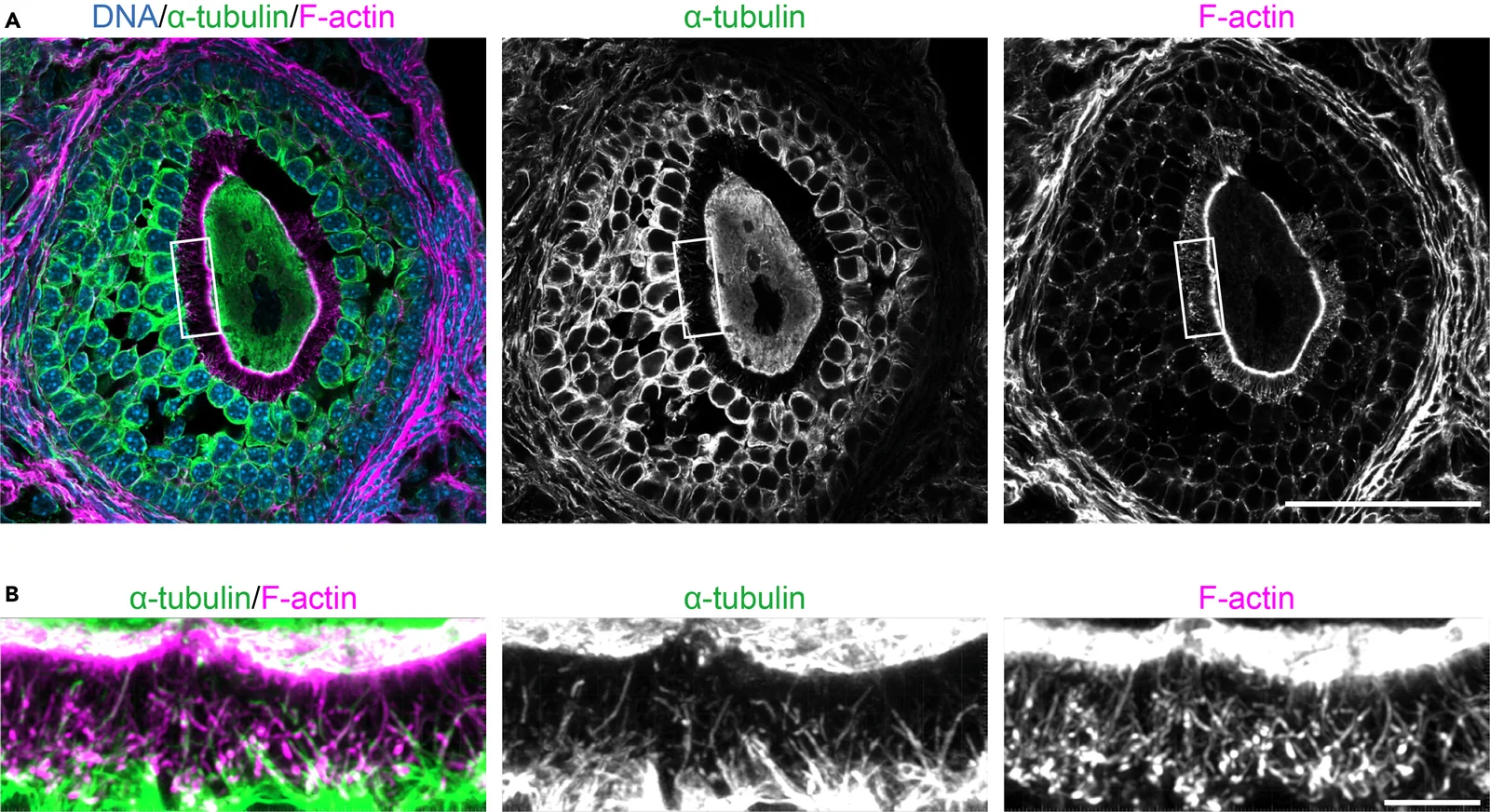
Representative immunofluorescence images of a secondary follicle and its transzonal projections (TZPs) in a mouse ovarian section (A) Immunofluorescence images of a secondary follicle stained for α-tubulin, F-actin, and DNA. Scale bar, 50 μm. (B) Front view of a three-dimensional reconstruction generated from z stack confocal images using Imaris, showing the granulosa cells, oocyte and TZPs. Scale bar, 5 μm.

The tissue fixation and staining procedures described in this protocol also have been successfully applied to mouse small intestine and kidney tissues.^2^^,^^3^ In these tissues, microtubules were similarly preserved and clearly visualized, demonstrating the broader applicability of the protocol for imaging microtubules in epithelial tissues.^2^^,^^3^

## Limitations

This protocol enables reliable preservation and visualization of microtubules within TZPs of mouse ovarian follicles. However, because the method relies on thin cryosections, only a portion of a follicle is captured in any given section. As a result, it is difficult to visualize all TZPs present within a single follicle or to reconstruct the complete three-dimensional organization of the TZP network. Researchers seeking to analyze the entire TZP population within individual follicles may require alternative approaches, such as serial sections imaging combined with 3D reconstruction.

In addition, because the protocol is performed on fixed tissue specimens, it provides only a static snapshot of microtubule organization. Analysis of microtubule dynamics in living follicles requires complementary live-cell imaging methods.

## Troubleshooting

### Problem 1

The ovaries do not sink in the sucrose solutions (Steps 7–9).

### Potential solution

Ensure that sufficient time has passed since immersion in the sucrose solutions. If the ovaries do not sink after three hours in 15% sucrose solution, overnight (approximately 12 h) in 20% sucrose solution, or five hours in 30% sucrose solution, it is possible that the fat was not adequately removed before tissue fixation. Before fixing the ovaries, the fat must be carefully removed under a stereomicroscope (Step 3). Ovaries containing excessive fat may remain floating despite prolonged incubation.

### Problem 2

The cells in the section are greatly distorted, or vacuoles form within the section (Step 16).

### Potential solution

• Ice crystal formation during freezing is a common cause of tissue distortion and vacuole-like artifacts. Make sure that the ovaries are sunk in the sucrose solution before freezing them (Steps 7–9).
• Frozen blocks that have been brought to −20°C for sectioning should not be brought back to −80°C (Step 15). It is possible that the moisture released from the sample refreeze and damage the tissue. If the sectioning cannot be completed, store the block at −20°C and section it as soon as possible.

### Problem 3

Microtubules within TZPs are not visible (Steps 5 and 35–43).

### Potential solution

• Confirm that PEM buffer was prepared correctly and has not deteriorated. If visible precipitates are present, prepare fresh buffer.
• Use freshly prepared fixative. For critical experiments, prepare the fixative from a freshly opened formaldehyde glass ampoule (Step 5).
• Ensure that fixation is initiated immediately after ovary dissection and performed for the recommended duration at 20–25°C (Step 5).
• Verify that the recommended fixative concentration and fixation time are used. Insufficient fixation may result in microtubule depolymerization, while excessive fixation may reduce antigen accessibility (Step 5).
• TZP microtubules are fine structures and may be more difficult to see than microtubules in granulosa cells. Optimize laser power and detector gain while avoiding detector saturation, and inspect the zona pellucida region using Airyscan images and z-stack datasets (Step 42).

### Problem 4

The background signal is high, resulting in poor image quality (Step 43).

### Potential solution

• Insufficient washing after staining can lead to a strong background signal. When washing, shake the slide well in a direction parallel to the slide (Step 29).
• Check the Airyscan detector view in Continuous mode to confirm that the detector is aligned. While imaging can still be performed even if the detector view remains red, the detector will not be able to perform at its full potential, resulting in a blurry image. Adjust the laser power and the detector gain until the detector view turns green (Step 42).

### Problem 5

The morphology and density of TZPs differ from the images shown in expected outcomes (Figures 3A and 3B; Step 43).

### Potential solution

The morphology and density of TZPs vary substantially during follicle development.^1^ Figures 3A and 3B show a representative secondary follicle and its TZPs. Later-stage follicles often exhibit reduced TZP density and more branched TZPs. Confirm the developmental stage of the follicle observed. In addition, the genetic background of mice may influence TZP morphology and density.

## Resource availability

### Lead contact

Further information and requests for resources should be directed to and will be fulfilled by the lead contact, Masamitsu Sato (masasato@waseda.jp).

### Technical contact

Technical questions on executing this protocol should be directed to and will be answered by the technical contact, Mika Toya (toya.mika.4v@kyoto-u.ac.jp).

### Materials availability

This study did not generate new unique reagents, and all materials in this study are commercially available.

### Data and code availability

• All data reported in this paper will be shared by the lead contact upon request.
• This paper does not report original code.
• Any additional information required to reanalyze the data reported in this paper is available from the lead contact upon request.

## Acknowledgments

We thank Junya Ito (Azabu University), Erina Kuranaga (Kyoto University), and Masatoshi Takeichi (RIKEN BDR) for helpful advice and discussions and for providing experimental materials. We also thank Kentaro Nakano (Tsukuba Univ.) for critical advice on fixation conditions that contributed to the development of this protocol. We thank SPF Administrative Office of Waseda University for animal care and husbandry. Cryosection preparation and Airyscan imaging were performed using research equipment (twins-01N301-f2, twins-B1C902-d1) at Waseda University, supported by the 10.13039/501100001700MEXT Project for promoting public utilization of advanced research infrastructure (program for supporting construction of core facilities; grant no. JPMXS0440500024). A.A. was supported by 10.13039/501100001695JST SPRING, grant no. B2R101263201. This work was supported by 10.13039/501100000646JSPS KAKENHI grant nos. 17K07397, 20K06645, and 25K09635 to M.T. and 16H04787, 18K19347, 23K27173, and 25H02582 to M.S. Support also came from the 10.13039/501100005927Daiichi Sankyo Foundation of Life Science to M.S., Ohsumi Frontier Science Foundation to M.T., and Waseda University grants for Special Research Projects grant nos. 2022C-170, 2023Q-012, 2024R-029 (to M.T.), 2017B-243, 2020R-038, 2023C-167, 2024C-490, 2025R-070, 2025C-154, and 2026C-507 (to M.S.).

## Author contributions

T.T. performed the experiments presented in this manuscript and prepared the initial draft of the manuscript. A.A. contributed to optimizing the protocol. M.T. developed the protocol, supervised the study, acquired funding, and wrote the manuscript. M.S. supervised the study, acquired funding, and reviewed and finalized the manuscript. All authors read and approved the final manuscript.

## Declaration of interests

The authors declare no competing interests.
