# Supplementary material for: Protocol for visualizing microtubules in transzonal projections of mouse ovarian follicles
Source: STAR Protoc. 2026 Oct 28;7(4):104924. doi: 10.1016/j.xpro.2026.104924 (PMC13735235; doi:10.1016/j.xpro.2026.104924)
Supplement: Supplementary file 1 [file mmc1.pdf]

Table S1: Image acquisition conditions of Figure 1A, Related to Step 38

Image Dimensions			
Scaling (per Pixel)	0.038 µm × 0.038 µm		
Image Size (Pixels)	3260 × 3260		
Microscope Information			
Microscope	Axio Observer.Z1 / 7		
Objective	Plan-Apochromat 40×/1.3 Oil DIC (UV) VIS-IR M27		
NA of objective	1.3		
Immersion	Oil		
Track and Detector Information			
Fluorophore / Target	Alexa Flour 647 (actin)	FITC (α-tubulin)	DAPI (DNA)
Pinhole	5.00 AU / 222 µm	5.00 AU / 169 µm	5.95 AU / 222 µm
Laser Wavelength	639 nm: 0.2%	488 nm: 0.6 %	405 nm: 0.6%
Scan Mode	Frame	Frame	Frame
Pixel Time	1.28 µs	1.28 µs	1.28 µs
LSM Scan Speed	5	5	5
Scan Direction	Bidirectional	Bidirectional	Bidirectional
Line Step	1	1	1
Averaging	1	1	1
Detection Wavelength	422-497,607-720	420-480,495-550	422-497,607-720
Imaging Device	Airyscan	Airyscan	Airyscan
Detector Type	GaAsP-PMT	GaAsP-PMT	GaAsP-PMT
Detector Gain	815 V	850 V	850 V
Detector Offset	0	0	0
Detector Digital Gain	1.0	1.0	1.0
Airyscan Mode	SuperResolution: 5.1 (2D SR, Auto)	SuperResolution: 4.9 (2D SR, Auto)	SuperResolution: 4.9 (2D SR, Auto)

Table S2: Image acquisition conditions of Figure 1B, Related to Step 42

Image Dimensions		
Scaling (per Pixel)	0.035 µm × 0.035 µm	
Image Size (Pixels)	718 × 287	
Z-Stack step size	0.15 µm	
Z-Stack range	3.15 µm (22 Slices)	
Microscope Information		
Microscope	Axio Observer.Z1 / 7	
Objective	Plan-Apochromat 63×/1.40 Oil DIC M27	
NA of objective	1.4	
Immersion	Oil	
Track and Detector Information		
Fluorophore / Target	Alexa Flour 647 (actin)	FITC (α-tubulin)
Pinhole	5.00 AU / 324 µm	5.00 AU / 247 µm
Laser Wavelength	639 nm: 0.4%	488 nm: 0.8%
Scan Mode	Frame	Frame
Pixel Time	1.19 µs	1.19 µs
LSM Scan Speed	6	6
Scan Direction	Bidirectional	Bidirectional
Line Step	1	1
Averaging	2	2
Averaging Mode	Line	Line
Averaging Method	Mean	Mean
Detection Wavelength	422-497,607-720	420-480,495-550
Imaging Device	Airyscan	Airyscan
Detector Type	GaAsP-PMT	GaAsP-PMT
Detector Gain	830 V	900 V
Detector Offset	0	0
Detector Digital Gain	1.0	1.0
Airyscan Mode	SuperResolution: 6.9 (3D, Auto)	SuperResolution: 6.7 (3D, Auto)
